# Dewetted Gold Nanostructures onto Exfoliated Graphene Paper as High Efficient Glucose Sensor

**DOI:** 10.3390/nano9121794

**Published:** 2019-12-16

**Authors:** Antonino Scandurra, Francesco Ruffino, Maria Censabella, Antonio Terrasi, Maria Grazia Grimaldi

**Affiliations:** 1 Department of Physics and Astronomy “Ettore Majorana”, University of Catania, via S. Sofia 64, 95123 Catania, Italy; francesco.ruffino@ct.infn.it (F.R.); maria.censabella@ct.infn.it (M.C.); antonio.terrasi@ct.infn.it (A.T.); mariagrazia.grimaldi@ct.infn.it (M.G.G.); 2 CNR-IMM, via S. Sofia 64, 95123 Catania, Italy

**Keywords:** glucose sensing, graphene paper, gold nanoparticles, thermal and laser dewetting

## Abstract

Non-enzymatic electrochemical glucose sensing was obtained by gold nanostructures on graphene paper, produced by laser or thermal dewetting of 1.6 and 8 nm-thick Au layers, respectively. Nanosecond laser annealing produces spherical nanoparticles (AuNPs) through the molten-phase dewetting of the gold layer and simultaneous exfoliation of the graphene paper. The resulting composite electrodes were characterized by X-ray photoelectron spectroscopy, cyclic voltammetry, scanning electron microscopy, micro Raman spectroscopy and Rutherford back-scattering spectrometry. Laser dewetted electrode presents graphene nanoplatelets covered by spherical AuNPs. The sizes of AuNPs are in the range of 10–150 nm. A chemical shift in the XPS Au4f core-level of 0.25–0.3 eV suggests the occurrence of AuNPs oxidation, which are characterized by high stability under the electrochemical test. Thermal dewetting leads to electrodes characterized by faceted not oxidized gold structures. Glucose was detected in alkali media at potential of 0.15–0.17 V vs. saturated calomel electrode (SCE), in the concentration range of 2.5μM−30 mM, exploiting the peak corresponding to the oxidation of two electrons. Sensitivity of 1240 µA mM^−1^ cm^−2^, detection limit of 2.5 μM and quantifications limit of 20 μM were obtained with 8 nm gold equivalent thickness. The analytical performances are very promising and comparable to the actual state of art concerning gold based electrodes.

## 1. Introduction

Glucose biosensors accounts for about 85% of biosensors industry. In fact, reliable and fast monitoring of glucose represents an important analytical tool in many scientific and technology fields such as health [1], food industry [2], environmental assessment [3], and molecular biology [4]. In particular, diabetes pathologies pushed the research to develop self-monitoring glucose and non-invasive glucose monitoring systems [5,6,7,8]. The electrochemical analytical method is attractive with respect to methods based on UV [9], plasmonic resonance [10], and surface enhanced Raman spectroscopies [11,12], on chromatography [13] and colorimetry [14], due to its easy fast monitoring, selectivity, sensitivity, simplicity of miniaturization and integration, stability, low-cost, and reliability [15]. Recently, the emergence of nanoscience and nanotechnology has led to great developments in electrochemical science and technology, which led to the new branch named electrochemical nanotechnology. This new branch of science and technology combines electrochemical techniques with nanomaterials to address important issues, particularly in the sensor science [16]. Many non-enzymatic electrochemical sensors based on nanostructured gold, nanostructured copper, or nickel oxide-hydroxide have demonstrated several advantages with respect to the enzymatic sensors, such as stability, low cost, and tolerance to numerous oxidizing or reducing chemical species co-existing in the analysis media [17,18,19,20,21]. Gold shows significant electro-catalytic properties against oxidation of many organic species [22]. In particular, it has been demonstrated that gold shows a high electro-catalytic activity towards glucose oxidation. Numerous papers have reported that nanostructured gold surfaces exhibit electro-catalytic activity towards oxidation of many organic species. Conversely, flat gold surfaces show poor or negligible catalytic properties [23,24]. The electro-catalyzed redox reactions by gold are highly pH dependent and are favored in alkali media, due to leading role of the negatively charged reaction intermediates. In fact, gold binds weakly the positively charged or neutral intermediates [25]. Nanostructured gold and nanocomposites based gold electrodes have been prepared using a variety of methods [26,27,28,29,30]. Gold nanostructures onto graphene and carbon nanotubes [31,32] have been constructed by chemical etching [33], electrochemical deposition in hydrophilic gel templates–gelatin and agarose [23], electrochemical etching [34], de-alloying [35], electrochemical deposition using gelatin template [36]. It is also pointed out the advantages of carbon materials used as substrate which include the possibility of a variety of chemical functionalization, the absence of interfering metal ions, low cost, high electrical conductivity, and large amplitude of working potential [37].

In our previous work, we reported the non-enzymatic detection of glucose and fructose by gold nanoparticles on graphene paper (GP-AuNPs) [38]. In particular, the sensitivity and detection limit of glucose detection are very encouraging and comparable or better than the actual state of art for gold based electrodes. The electrodes were prepared by fully dry processes based on laser or thermal dewetting of a gold layer. Our processes are characterized by two fully dry steps: gold deposition by sputtering and laser or thermal dewetting. Conversely, other methods consist of multi steps solution based processes for the preparation of gold nanostructures, often in presence of an enzyme. The proposed electrodes are biocompatible thanks to the absence of poisonous metals such as copper or nickel.

In order to shed light on the synergistic effects observed in the electro-catalysis of glucose oxidation by gold nanostructures on exfoliated graphene paper, in the present work we investigated deeply the exfoliation phenomena and the surface chemical composition of gold nanostructures obtained by laser or thermal dewetting. Moreover, the stability and the morphology modification of both laser and thermal dewetted gold nanostructures under glucose test conditions are presented and discussed.

## 2. Materials and Methods

### 2.1. Materials

Potassium ferrocyanide, potassium chloride, D-(+)-glucose, phosphate buffer solution at pH 12, graphene paper foil 240 µm thick (XG Sciences), were purchased by Merck, Rome, Italy. All aqueous solutions were made by ultrapure water.

### 2.2. Electrode Fabrication

**Figure 1:**
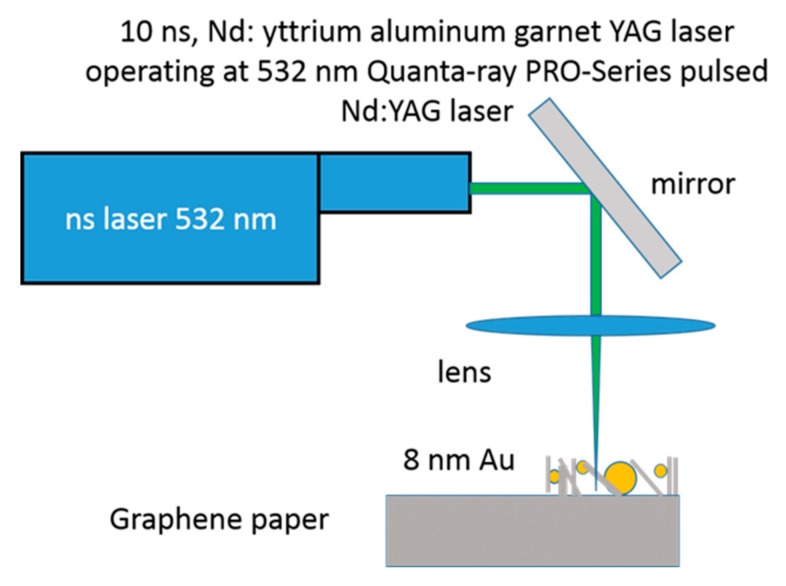
(color online) Scheme of the laser irradiation experiments.

Pieces of graphene paper of measuring 1 × 2.5 cm and 240 µm thick were used. The function of graphene paper was of support, electrical conductors and active material of the electrode. Au films (1.6 or 8 nm thick) were sputtered onto 1 × 1 cm of the support area, leaving the remaining surface not metallized. The gold films were deposited by sputtering using the Emitech K550X sputter coater. The conditions of 50 mA, 40 s and 10 mA, 60 s of plasma current and time deposition were used for 8 nm and 1.6 nm of Au, respectively. Molten-phase dewetting of gold layer was obtained by nanosecond laser annealing using a pulsed (10 ns) Nd: yttrium aluminum garnet laser operating at 532 nm (Figure 1). To irradiate surface area of 1 × 1 cm on the sample, several nearby single beam spots were overlapped.

### 2.3. Instrumental Characterizations

Raman characterization of graphene substrate was obtained by the Horiba Jobin Yvon IHR 550 spectrometer, equipped with the laser source at 632.81 nm. The analysis spot was of 100 μm. The wavelength scale was referenced to the Si czochralski peak at 520.8 cm^−1^. The electrochemical characterization of graphene paper was obtained at room temperature in unstirred and not deaerated solution of ferrocyanide probing molecule at 10^−3^ M in KCl 1 M supporting electrolyte at a scan rate of 0.02 Vs^−1^. The potentiostat Versastat 4 by Princeton Applied Research was used. Saturated Calomel Electrode (SCE) and Pt electrode were used as reference and counter electrode, respectively. Cyclic voltammetry and amperometric responses were used to investigate the electro-catalytic properties of GP-AuNPs towards glucose oxidation. Before use, the electrodes were electrochemically cleaned by 5 cycles between −0.5/+1 V, in phosphate buffer solution at pH 12. Cyclic voltammograms were obtained at a scan rate of 0.020 V s^−1^. The morphology was investigated by SEM by a Gemini 152 field emission Carl Zeiss Supra 25. The analyses were performed with an acceleration voltage of 5 kV with an aperture size of 30 μm, a working distance of 4–5 mm, and using an In-lens detector. XPS was employed to investigate the surface and the carbon-gold interaction in laser and thermal dewetted sensors. XPS spectra were obtained using a Mg kα photon source (1253.6 eV) and a hemispherical electron analyzer VG Microtech CLAM4 equipped with a multi-channeltron detector (MCD). RBS was used to quantify the gold in GP-AuNPs electrodes. The analyses (2.0 MeV He^+^ beam, normal incidence) were performed by detection at 165° backscattering angle, using a 3.5 MV HVEE Singletron accelerator system.

## 3. Results and Discussion

### 3.1. Morphological and Structural Characterization of Laser Irradiated Graphene Paper 

**Figure 2:**
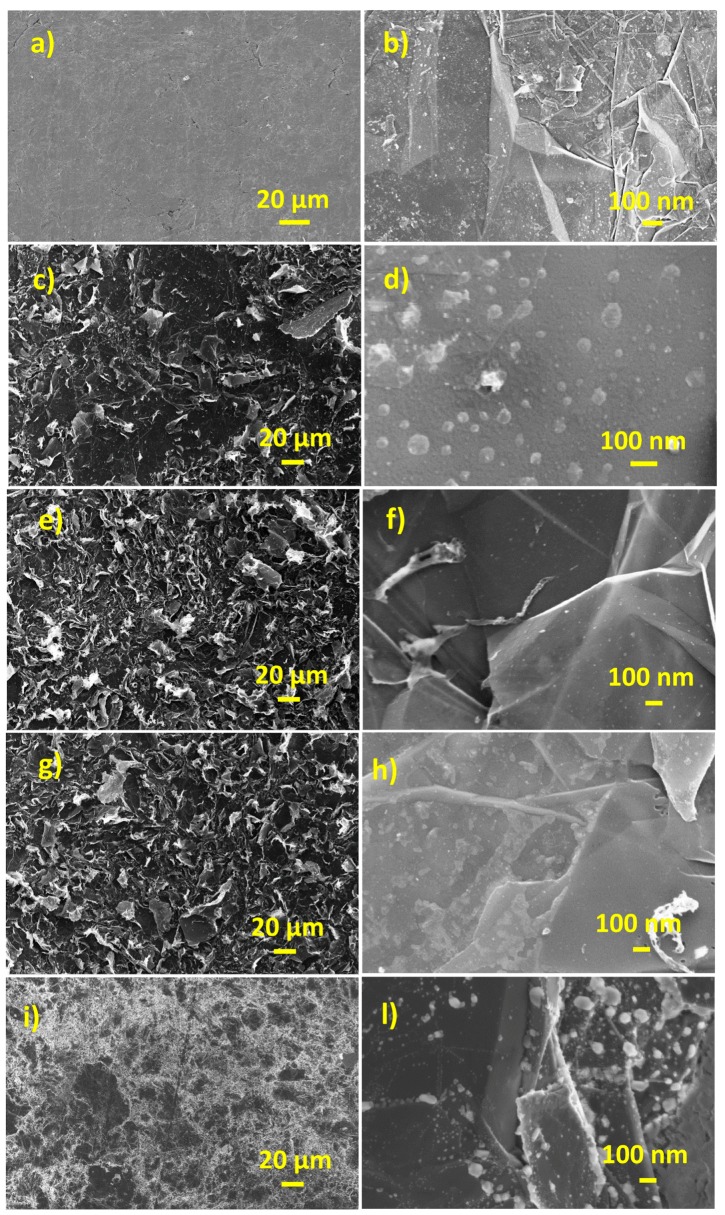
(color online) SEM micrographs of graphene paper (**a**,**b**) pristine; laser irradiated at fluence of (**c**,**d**) 0.25 Jcm^−2^; (**e**,**f**) 0.5 Jcm^−2^; (**g**,**h**) 1.5 Jcm^−2^; (**i**,**l**) thermal treated at 400 °C, 1 h in N_2_.

Figure 2 reports typical SEM pictures of pristine graphene paper (a,b) and after laser irradiation at fluence of 0.25 Jcm^−2^ (c,d), 0.5 Jcm^−2^ (e,f) and 1.5 Jcm^−2^ (g,h), respectively. The picture of pristine sample shows the presence of flat layers differently oriented on the plane. In addition, defects consisting of cracks and voids are present. The laser irradiation induces the graphene paper exfoliation, visible by the presence of numerous nanosheets. Moreover, some bright spots with a size of about 50 nm can be observed. These spots can be ascribed to the presence in some regions of surface blistering. No significant differences have been found within the explored fluence values. Thermal treatment at 400 °C of graphene paper does not produces significant modifications of the morphology (Figure 2i,l).

**Figure 3:**
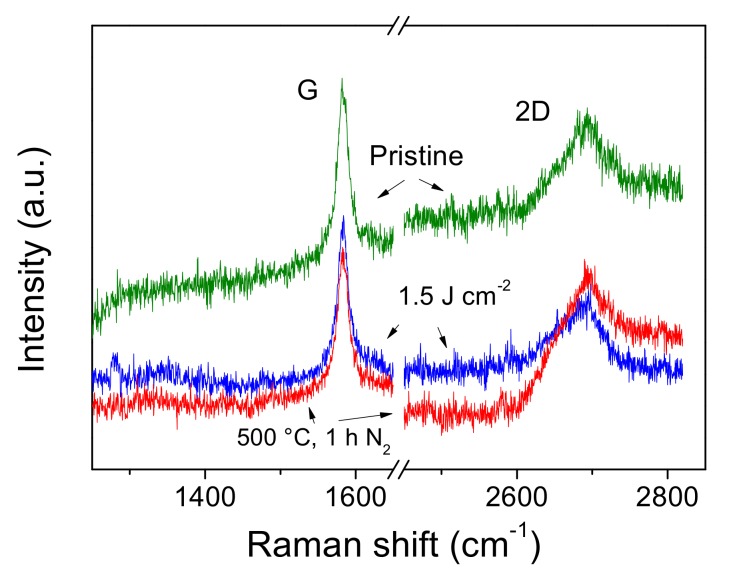
(color online) Micro Raman spectra of pristine graphene paper and after laser irradiation at 1.5 Jcm^−2^ of fluence and treatment at 500 °C 1 h, N_2_.

Figure 3 reports typical micro Raman spectra of untreated (pristine) and laser irradiated graphene paper at fluence of 1.5 Jcm^−2^. The spectrum of untreated sample shows a first order peak centered at 1581.4 cm^−1^, assigned to G-peak, and a second order broad signal centered at about 2689.9 cm^−1^, which is assigned to 2D-peak. The spectra of thermally treated samples in the range of 300–500 °C and laser treated samples in the range of fluence 0.25–1.5 Jcm^−2^ show similar signals. Figure 3 reports the spectra corresponding to the most severe stress conditions studied, i.e., 1.5 Jcm^−2^ e 500 °C. Therefore, the above corresponding assignments valid for pristine sample can be still applied for the treated samples. Basing on the Raman characterization, we excluded the presence of significant structural disorder of the substrate in the explored conditions [39]. XRD patterns of both pristine and laser irradiated samples (data not shown) are characterized by only one peak at 2ϑ = 26.54°. This signal represents the main graphite contribution (symmetric group P63/mmc) assigned to the (0002) crystallographic planes. Accordingly, graphene paper substrates consist of multiple layers of graphene sheets, with a high degree of structural order.

### 3.2. Electrochemical Characterization of Graphene Paper Modified by Laser Irradiation

**Figure 4:**
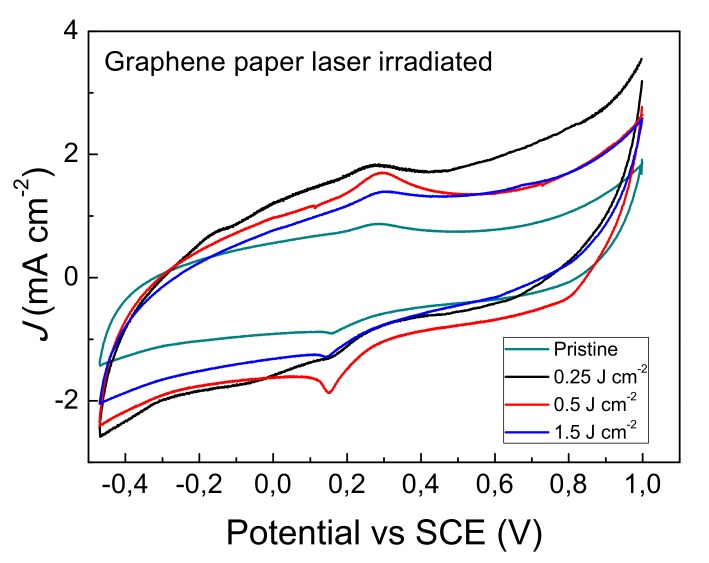
(color online) Cyclic voltammograms of graphene paper as received and after laser irradiation recorded at 0.02 V s^−1^, obtained using Fe(CN)_6_^3^^−^/FeCN)_6_^4^^−^ 1 mM in KCl 1 M as supporting electrolyte.

Carbon materials, conversely to other substrates, reduce the interference in the detection of the analyte. In particular, graphene-based materials offer high electrical conductivity and large electrochemical working potential [40]. The exfoliation of graphene paper, induced by laser irradiation, and the subsequent formation of nanosheets increase the real area of the electrode. The electrochemical characterization of laser modified graphene paper was carried out by cyclic voltammetry, using ferricyanide complex as probing molecule. The electro-active surface area *A_R-S_*, the heterogeneous rate constant *k*^0^ and the capacitance *C* of electrode-solution interface were determined by cyclic voltammetric curves. Figure 4 reports typical cyclic voltammograms of pristine graphene paper (untreated) and after laser irradiation at 0.25, 0.5, and 1.5 Jcm^−2^. Voltammograms are characterized by the presence of ferrocyanide anodic and cathodic peaks, which position is influenced by the laser surface treatment. The two peaks are superimposed to significant capacitive current. The high electrode-solution capacitance is likely produced by potassium ions intercalation in between graphene planes. The intercalation is favored by the applied potential and proceeds via adsorption of potassium ion on the graphene paper surface and absorption of potassium ion below the top graphene layer, then further exposure to potassium ions leads the intercalate structure [41]. Voltammograms curves shown in Figure 4 were employed for the quantitative analysis of *A_R-S_*, *k*^0^, and *C*. The active electrode area was determined using the Randles-Sevčik equation valid in quasi-reversible electron transfer processes [42]:

(1) $$I_{p}=2.686\;\times 10^{5}\;\times \;\sqrt[{2}]{n^{3}}\;A_{R-S}\;C\;\sqrt[{2}]{D\nu }$$

where *n* is the number of electrons participating in the redox process (*n* = 1 for ferricyanide), *D* is the diffusion coefficient (7.6 × 10^−6^ cm^2^ s^−1^) [43], *C* (10^−6^ mole cm^−3^) is the concentration of the probe molecule, ν is the scan rate (0.02 Vs^−1^) and $I_{p}$ is the anodic peak current. According to the experimental conditions adopted by us, the working area was calculated using the following Equation (2):

(2) $$A_{R-S}\;\left[cm^{2}\right]=\;\frac{I_{p}\;\left[A\right]}{1.0475\;\times 10^{-4}}$$

The heterogeneous rate constant, *k*^0^, was estimated using the Nicholson model [44]:

(3) $$k^{0}=\;Ѱ\;\sqrt{\frac{\pi nF\nu Dox}{RT}}\;{\left(\frac{Dox}{Dred}\right)}^{-\left(\frac{nF\nu }{2RT}\right)}$$

In Equation (3), the heterogeneous rate constant *k*^0^ is related to (Ѱ), which is a kinetic parameter depending on the anodic-catodic peak separation ΔEp (Ea-Ec), according to the working curves [44]. Dox is the diffusion coefficient of the potassium ferricyanide, Dred is the diffusion coefficient of the potassium ferrocyanide Fe(CN)_6_^4−^ (6.3 × 10^−6^ cm^2^ s^−1^) [43], α is the transfer coefficient (nFν/RT = 0.78), while *R*, *T*, *n*, *F* represent the universal gas constant, the absolute temperature, the number of electrons involved in the redox reaction, and the Faraday constant, respectively. Basing on our experimental conditions, the heterogeneous rate constant *k*^0^ was calculated according to the following Equation (4):

(4) $$k^{0}=\;Ѱ\;\times \;4\;\times \;10^{-3}$$

Table 1 reports the data of the various parameters obtained for the graphene paper based electrodes. The real electro-active area, Areal, which furnish information on active surface area and on the presence of inactive regions over the electrode area, was calculated from the following Equation (5):

(5) $$A_{\mathrm{real}}=\frac{A_{R-S}}{A_{\mathrm{geo}}}$$

where *A_geo_* is the geometric area of the electrode. *A_real_* is described also as the ‘roughness factor’. The graphene paper reference shows a high real area which is much greater with respect to that of screen printed carbon paste electrodes, typical of about 0.6 [45]. The laser irradiated samples show an increase of the real area which reach a maximum in the case of a fluence of 0.5 Jcm^−2^. This result is in agreement with SEM analyses that revealed the presence of numerous flakes on the surface, as consequence of graphene paper exfoliation. Moreover, the trend observed for the real area in laser irradiated samples maybe due to balancing effects of interface air (that decreases) and of graphene edges (that increase) the number of sites for the electron transfer of the Fe(CN)_6_^3−^ + 1 e− ⇄ Fe(CN)_6_^4−^ reaction.

The peak separation, which is related to the reversibility of the electrochemical process, is low for untreated graphene paper and increases with the fluence. Accordingly, the heterogeneous rate constant *k*^0^ decreases for irradiated samples with respect to the untreated, indicating a raising of the irreversibility of the process. This finding could be attributed to the partial oxidation of the laser irradiated surfaces. Another interpretation may be based on the presence of very thin air layer trapped at the electrode-solution interface, which is formed for the presence of graphene flakes structures. Moreover, the air trapped at electrode-solution interface maybe the consequence of reduced wettability of highly wrinkled surface. 

The capacitance of electrode-solution interface has been calculated using the following equation:

(6) $$C=\frac{\int_{E_{1}}^{E_{2}}i\left(E\right)dE}{2\nu \left(E_{2}-E_{1}\right)}$$

where *ν* is the scan rate dV/dt, *i*(*E*) is the instantaneous current, $\int_{E_{1}}^{E_{2}}i\left(E\right)dE$ is the absolute area obtained by integration of positive and negative portion of voltammogramm, except the faradic peak portions. (*E*_2_ − *E*_1_) is the potential width. The capacitance of the electrode-solution interface, that furnish an indication on the electrode-solution interface extension, increases in laser irradiated samples. However, slight diminution with fluence on going from 0.25 to 1.5 Jcm^−2^ can be observed. This last experimental finding does not have a straightforward explanation, nevertheless air trapping at the highly wrinkled electrode-solution interface could influence the capacitance. The parameters reported in Table 1 and the size distribution of gold nanoparticles (see next paragraph) were used for the selection of the optimal laser fluence in the realization of the GP-AuNPs sensors and for further investigations.

### 3.3. GP-AuNPs Morphology Characterization

**Figure 5:**
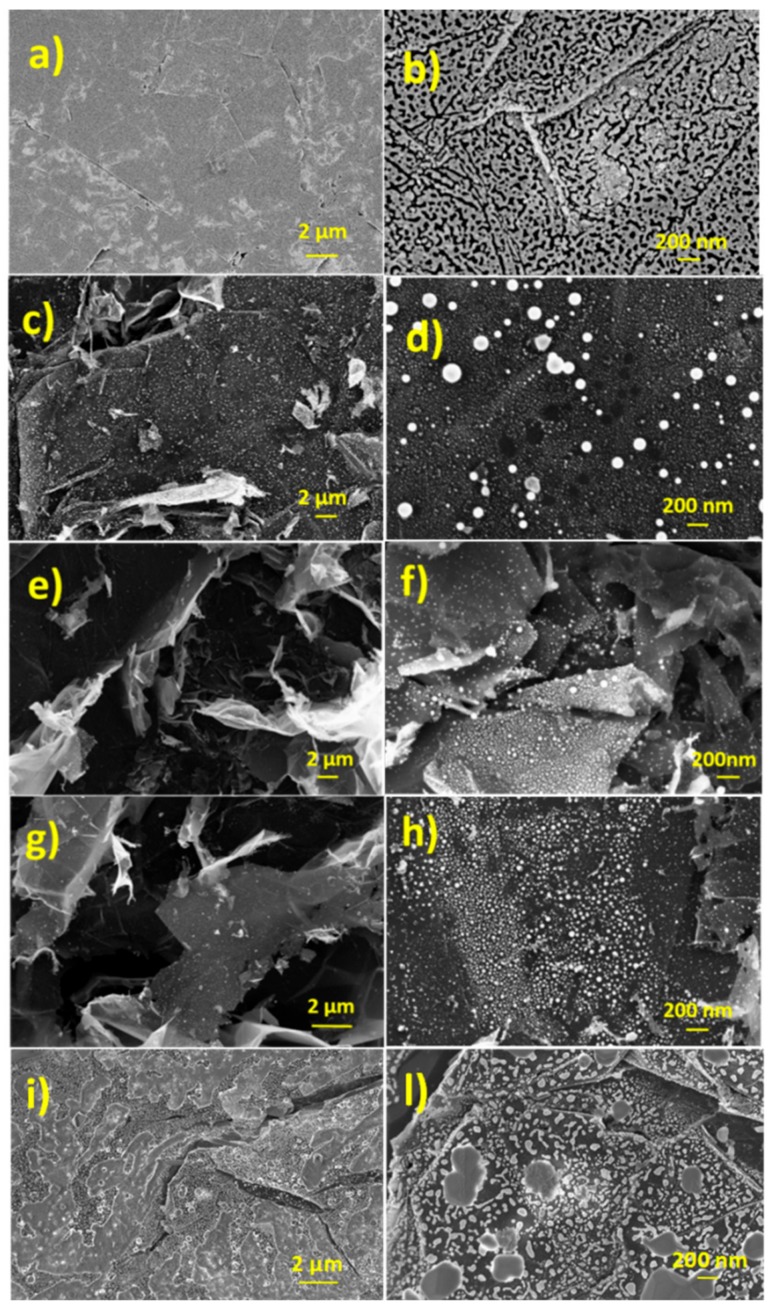
(color online) SEM pictures of (**a**,**b**) Au 8 nm layer as deposited onto graphene paper and after: laser dewetting at fluence of (**c**,**d**) 0.25 Jcm^−2^; (**e**,**f**) 0.5 Jcm^−2^; (**g**,**h**) 1.5 Jcm^−2^; (**i**,**l**) thermal treatment at 400 °C, 1 h in N_2_.

The surface morphology of gold layer deposited onto graphene paper was analyzed by SEM at two magnifications. The corresponding pictures are shown in Figure 5a,b. The surface morphology is conformal to that of the graphene paper (see Figure 2a). A fine structure with the presence of holes can be observed in the Figure 5b. The gold morphology is representative of the nucleation mechanism observed in the early stages of thin films growth at room temperature onto not wettable substrates [46]. Figure 5c,h shows typical SEM pictures of the gold layer after the laser dewetting at fluence of 0.25, 0.5 and 1.5 Jcm^−2^, respectively. Laser dewetted surfaces consist of exfoliated graphene paper with spherical AuNPs composites. The size distribution of AuNPs range between 10 to 150 nm. Increasing the laser fluence ongoing from 0.25 to 1.5 Jcm^−2^ the size distribution goes towards smaller size. 

For comparison, we studied the effects of the thermal dewetting. Figure 5i,l shows typical SEM pictures of graphene-gold nanostructures obtained by thermal dewetting at 400 °C. We observed that dewetting conditions in the temperature range of 300–500 °C gave similar gold nanostructures. Typical gold faceted nanostructures characterized by wide distribution in the range 200–400 nm where observed. Accordingly, the main difference in the morphology exhibited by the thermal and laser dewetting processes consist in the size distribution and shape of the resulting nanoparticles [47]. Nanosecond laser dewetting induces an increase of temperature that, depending on pulse power and duration, heat loss and substrate thermal diffusivity, can reach 2000 K [47]. This temperature is above the gold melting point (1337.33 K) [48], thus the obtained nanoparticles are almost spherical. Below the melting point, dewetting of gold on graphene paper occurs at solid state to minimize the total surface free energy, since the system is thermodynamically unstable at elevated temperature.

Basing on the AuNPs size distribution and on the active area of the graphene paper after laser irradiation, we chose the fluence of 0.5 Jcm^−2^ for the further investigations.

### 3.4. XPS Characterization of GP-Au Nanostructures

**Figure 6:**
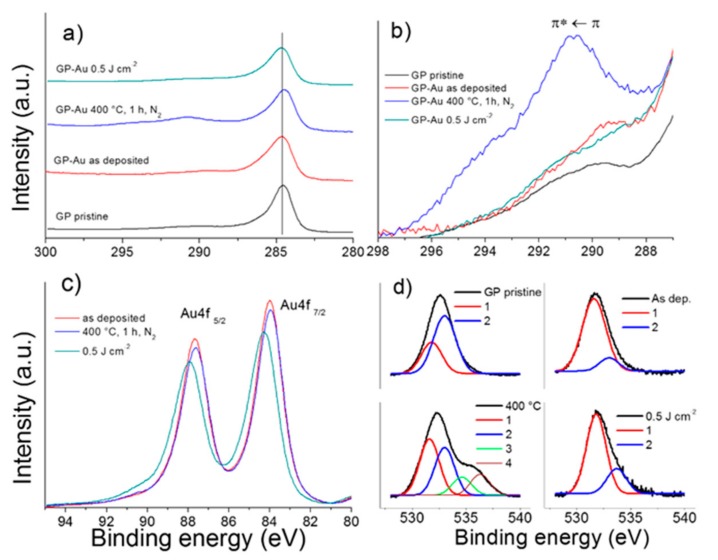
(color online) (**a**) XPS C1s core-level spectra of pristine GP and GP-Au as deposited and after 400 °C thermal dewetting and 0.5 Jcm^−2^ laser dewetting. The vertical line has the function to facilitate visualization. (**b**) enlarged spectral regions showing the satellite structure normalized to the C1s peak, after linear background subtraction; (**c**) corresponding Au4f spectra; (**d**) corresponding O1s spectra.

XPS analyses were performed on pristine graphene paper (GP) and on gold metallized electrodes. The survey spectra (not reported) show the signals of carbon, oxygen and gold. Figure 6a reports the C1s core-level spectra of pristine GP and of GP-Au as deposited and after 400 °C thermal and 0.5 Jcm^−2^ laser dewetting. The main line is centered at 284.5–284.7 eV and is characterized by high asymmetry, largely because the material is good electric conductor [49]. An additional contribution to the asymmetry originates from the presence of C1s component at about 286.6 and 288 eV (deconvolution not shown) that are assigned to C–O and C=O functional groups, respectively. The features of the main line are in agreement with *sp*^2^ hybridized carbon atoms, associated to π type valence electrons free-like present in the GP. On the high binding energy side of the C1s spectra, π type shake-up satellites are present. Figure 6b reports the enlarged spectral regions of these satellite structures normalized to the intensity of the C1s main peak, after linear background subtraction. In the spectra of pristine GP and as deposited GP-Au the satellite features are observed at about 5 eV from the core-level peaks, which are assigned to the π *← π transition. Similar shake-up structure was found at 4.4 eV in the case of HOPG [50]. In the thermal and laser dewetted electrodes, the main satellites are found at 6 and 6.4 eV respectively, from the core-level peaks. These satellites are assigned the dipole like π *← π shake-up transition (ΔL = 1). The significant intensity and the binding energy shift of the shake-up features observed in the spectra of dewetted samples can be attributed, at least in part, to the interaction of π valence electrons with plasmon excitations in the nanostructure form of gold. Additional satellite with minor intensity was found at 10 eV from the main peak. The spectrum of laser dewetted sample shows a less intense shake up feature that can be attributed to a partial loss of π conjugation of the outermost graphene layers. Figure 6c reports the corresponding Au4f-core level spectra. The Au 4f_7/2_ peak is centered at 84 eV in the case of as deposited and thermal dewetted samples, which is assigned to metallic gold Au(0), whereas the spectrum of laser dewetted sample shows the peak centered at 84.2–84.3 eV which can be assigned to Au(I), likely in the form of Au-O, as consequence of partial gold nanoparticles oxidation [51]. Figure 6d reports the corresponding O1s core-level spectra. The spectra were deconvoluted using Gaussian components centered at 531.6–531.8 and 533 eV (Gaussians 1–2), which are assigned to C=O and C–O groups, respectively. Moreover, the spectrum of thermal dewetted electrode shows two additional components centered at about 534.6 and 536.3 eV, respectively (Gaussians 3–4), that can be assigned to adsorbed water and adsorbed oxygen [52]. The component in the O1s spectrum of laser dewetted electrode that would be assigned to Au-O, which is expected weak and centered at 529.4 eV [53], maybe masked by the tail of the components at 531.8 eV. For these reasons, it was not distinguished in the spectrum. It has been proven that gold in the form of nanoparticles can be easily oxidized, still maintain the high catalytic activity toward oxidation of organic molecules [53]. Table 2 reports typical quantitative surface compositions of the as deposited GP-Au and thermal and laser dewetted electrodes measured by XPS. The amount of gold detected on the surface of dewetted electrodes is lower than that deposited, according with the fact that dewetted gold exposes less surface in order to minimize the surface energy.

### 3.5. Glucose Sensing

**Figure 7:**
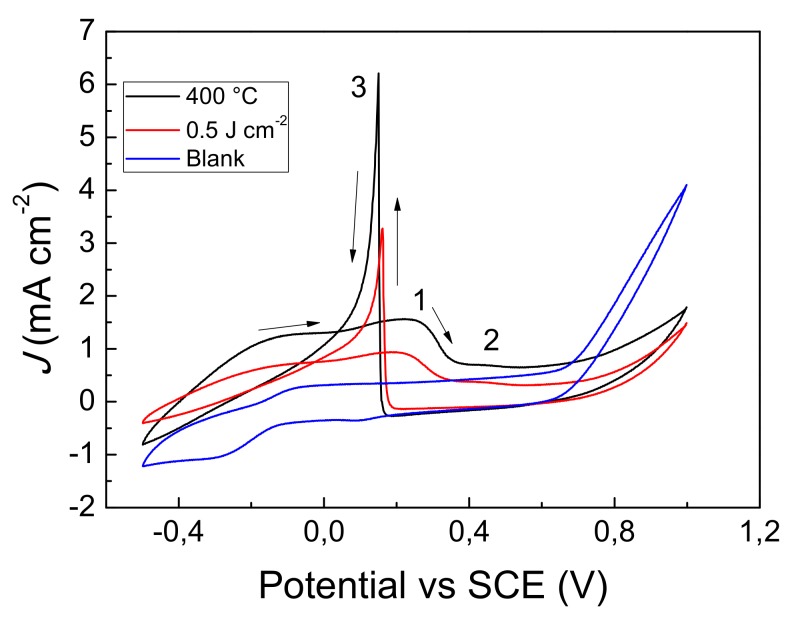
(color online) (left side) Cyclic voltammograms of glucose 30 mM detected by GP-AuNPs electrodes obtained by thermal or laser dewetting.

The electro-catalytic activity of the GP-AuNPs sensors was studied by cyclic voltammetry. Figure 7 reports the voltammograms obtained by the 400 °C thermal and 0.5 Jcm^−2^ laser dewetted sensors. Both voltammograms in the forward scan shows two broad peaks at 0.25 V (1) and 0.45 V (2), respectively. The peak at 0.25 V is assigned to the formation of gold hydroxide and the adsorbed glucose intermediate is oxidized to gluconolactone. At more positive potentials, we found the weak peak at 0.45 V which is assigned to the oxidation of the gold hydroxide layer, that became oxidized to gold oxide. At this stage the glucose oxidation is inhibited. In the backward scan, the gold oxide was reduced to gold hydroxide, which electro-catalyzes the glucose oxidation through the two electron process. This process is evidenced by the intense and sharp anodic peak (3) observed at 0.17 V for the laser dewetted and at 0.15 V for the 400 °C thermal dewetted sensor, respectively. According to the XPS data, the observed shift could be attributed to the different chemical state of gold found in the two sensors. 

**Figure 8:**
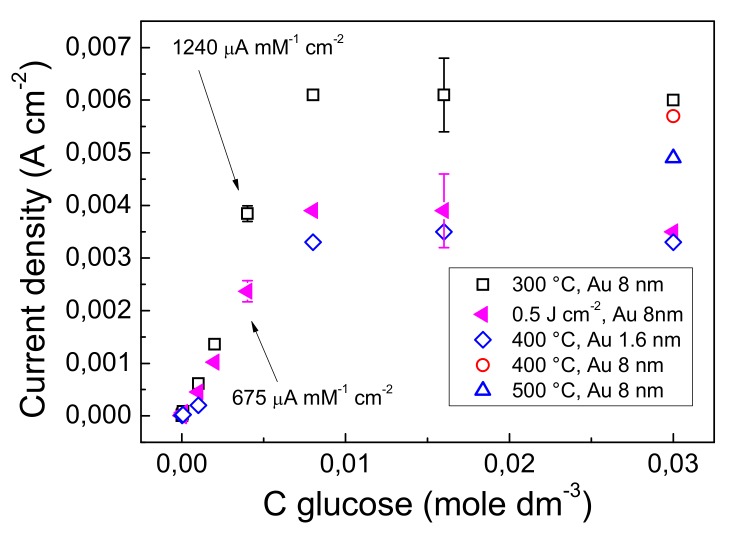
(color online) Current calibration of glucose detected by GP-AuNPs electrodes obtained by thermal or laser dewetting.

Current calibration curves for glucose detection are reported in Figure 8. The curves were obtained at a potential of 0.15 V (thermal electrodes) and 0.17 V (laser electrode). The response to glucose was determined in the concentration range of 2.5 µM to 30 mM. There is little influence of dewetting temperature in the range 300–500 °C on the electrode response. In this temperature range there is no significant variation of gold content (see paragraph 3.6). Moreover, Figure 8 reports the response of the sensor obtained by dewetting at 400 °C a gold layer 1.6 nm thin. The thickness of 1.6 nm was chosen to have a final average gold quantity comparable to that of the 0.5 Jcm^−2^ laser dewetted sensor (see paragraph 3.6). The response to glucose of the 400 °C 1.6 nm gold sensor was found similar to that of the 0.5 Jcm^−2^ laser dewetted sensor obtained starting from 8 nm gold. Based on this finding, the amount of gold present in the form of nanostructures on the sensor surface determines the electrode response. In fact, gold is not just a catalyst, but participate in the oxidation reaction of glucose. The responses of the electrodes reflect the gold concentrations at surface reported in Table 2. Accordingly, the apparent lower response of the laser dewetted electrode can be attributed to the lower gold content. The presence of Au(I) in the laser dewetted electrodes seems to influence the peak position of the two-electron oxidation, but not the sensor current response.

Quasi-linear current response was obtained by both type of electrodes in the concentration range from 20 µM–8 mM. Conversely, a nonlinear response was observed in the range of concentration 8–30 mM. The absence of linearity in latter concentration range is caused by the diffusion controlled mechanism that limits the concentration of glucose on the sensor surface. Sensitivities of 1240 µA mM^−1^ cm^−2^, and of 675 µA mM^−1^ cm^−2^ were obtained in the concentration range of 2–4 mM by the 300 °C thermal and 0.5 Jcm^−2^ laser dewetted electrodes, respectively. A detection limit of 2.5 μM and quantification limit of 20 μM were obtained. The data are competitive with respect to the state of the art of recent literature results concerning various non-enzymatic/enzymatic hybrid and gold-based nanostructures for glucose biosensors [20,28,54,55,56,57]. The interference of the common species such as ascorbic acid and uric acid was studied and the details were reported elsewhere [38]. The observed interference effects are mainly attributed to the adsorption of UA that dirty the electrode surface.

### 3.6. Electrode Stability

**Figure 9:**
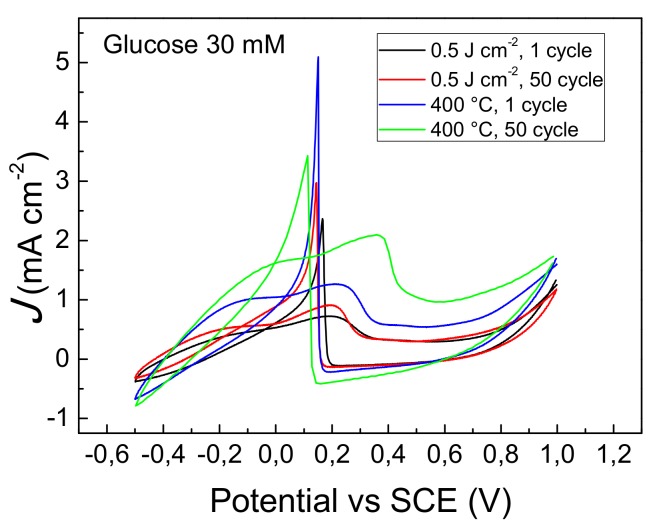
(color online) Cyclic voltammograms of glucose 30 mM detected by GP-AuNPs electrodes thermally and laser dewetted at fluence of 0.25 Jcm^−2^. Comparison of the 1st and the 50th cycles.

**Figure 10:**
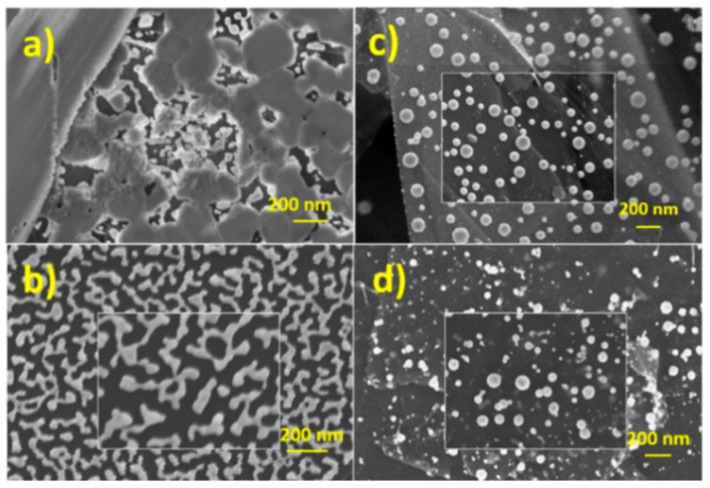
(color online) SEM pictures of electrodes prepared by thermal dewetting at (**a**) 400 °C and (**b**) after 50 voltammogram cycles between −0.5 to 1 V; (**c**) laser dewetting at 0.5 Jcm^−2^ of fluence and (**d**) after 50 voltammogram cycles between −0.5 to 1 V.

**Figure 11:**
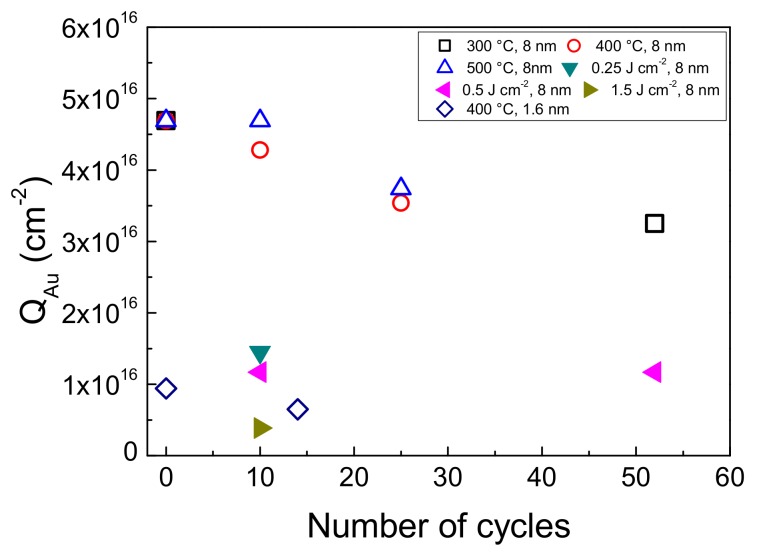
(color online) Gold content measured by RBS as function of initial thickness and the number of valtammogram cycles (buffer solution at pH 12) for: 8 nm layer thermal dewetted at 300 °C, 400 °C and 500 °C; 1.6 nm layer thermal dewetted at 400 °C; 8 nm layer laser dewetted at 0.25, 0.5 and 1.5 Jcm^−2^ of fluence.

The stability of laser dewetted in comparison to the thermal dewetted sensors was studied at pH 12 employing glucose solutions at different concentrations. Figure 9 shows the most significant modification of electrode response observed by glucose solution 30 mM. In the Figure the 1st and the 50th cycles of voltammograms registered by GP-AuNPs sensors dewetted at 400 °C and at fluence of 0.25 Jcm^−2^ respectively, are compared. The laser dewetted electrode shows almost identical voltammograms, indicating a huge stability under the adopted conditions, whereas the thermal dewetted electrode shows a modification of the shape of the voltammogram. In particular, the two electron oxidation peak is reduced in intensity and sharpness and shifted to lower potential on going from the 1st to the 50th cycle. Simultaneously, an increase of the peak at 0.45 V was observed. These findings can be ascribed to a reduced and/or modified electro-catalytic property of the gold nanostructures. Figure 10a,b show the SEM pictures of the sensor obtained by thermal dewetting at 400 °C before and after 50 voltammogram cycles between −0.5 to 1 V, respectively. The morphology of the gold faceted nanostructures is clearly heavily modified. Conversely, the laser dewetted sensor at 0.5 Jcm^−2^ of fluence shows almost unmodified morphology after 50 voltammogram cycles between −0.5 to 1 V (Figure 10c,d). According to XPS data, the high stability of laser dewetted electrode can be attributed to the presence of Au(I) in the AuNPs. The formation of bonds with oxygen blocks the self-diffusion of the gold atoms and preserves the nanostructures. Figure 11 reports the gold content measured by RBS as function of voltammogram cycles for various electrodes. In thermal dewetted electrode the gold content decreases with the number of voltammogram cycles. This is related to a partial gold dissolution during the cycles. Conversely, laser dewetted electrodes show an almost unmodified gold content. The gold content in laser dewetted electrodes is related to the fluence, and is reduced by partial laser ablation. 

## 4. Conclusions

In this work we described and characterized two simple processes to obtain low cost, high throughput gold nanostructures on graphene paper for high sensitivity glucose detection. The electro-catalytic oxidation of many organic compounds, including sugars, is efficient on surfaces of nanostructured gold. The efficiency of these reactions starts when gold nanoparticles dimensions go to a nanometer scale. Despite the numerous currently experimental and theoretical studies, the mechanisms of electro-oxidation of glucose on gold are still not fully understood. The electro-catalytic oxidation of glucose on gold surface is highly pH dependent, and is favored in alkali media. This has been interpreted considering the significant role of negatively charged reaction intermediates, such as the crucial step of OH^−^ adsorption. Graphene based support can act synergistically with gold nanoparticles, enhancing the sensitivity and selectivity of gold alone. This enhancement can be interpreted by the efficient charge transfer from the Au nanostructures to the π delocalized orbitals in the graphene paper. This phenomenon maybe at the basis of the significant sensitivity of the proposed sensors. The electrode sensitivity is related to the total amount of gold present on the surface, according to the fact that gold is not just a catalyst, but participate to the oxidation reaction of glucose. The exfoliation of graphene paper produced by nanosecond laser irradiation leads to an increase of the electrode real area up to about a factor two. This phenomenon increases further the sensor analytical performances. XPS analyses of laser dewetted electrodes evidenced the formation of gold oxide. Oxidized gold nanoparticles are characterized by high morphology stability under the alkali glucose detection test and unmodified electro-catalytic activity.

## Figures and Tables

**Table 1:** Electrochemical parameters of laser irradiated graphene paper obtained by cyclic voltammetry.

Sample	Working Area^(1)^ *A_R-S_* (cm^2^)	*A_real_* = *A_R-S_*/*A_Geom_*	Δ*E*p = *E*a − *E*c (mV)	*k*^0^ (×10^−3^) (cm s^−1^)	C (mF cm^−2^)
Pristine	0.764	1.70	84.4	4	35.3
0.25 Jcm^−2^	0.477	1.91	101	2	69.1
0.5 Jcm^−2^	0.954	3.82	128.8	1.2	60.7
1.5 Jcm^−2^	0.582	2.32	134	1.1	53.3

^(1)^ calculated by the Randles-Sevčik equation using anodic peak current.

**Table 2:** Elemental composition of gold metallized electrodes (Atomic concentration %).

Sample	Au	C	O
As deposited	12.3	77.4	10.3
400 °C, 1 h, N_2_	5.3	67.3	27.4
0.5 Jcm^−2^	3.9	86.5	9.6
